# Supplementary material for: Epithelial-to-Mesenchymal Transition Mediates Resistance to Maintenance Therapy with Vinflunine in Advanced Urothelial Cell Carcinoma
Source: Cancers (Basel). 2021 Dec 12;13(24):6235. doi: 10.3390/cancers13246235 (PMC8699401; doi:10.3390/cancers13246235)
Supplement: Supplementary file 1 [file cancers-13-06235-s001.zip › cancers-1469087-supplementary.pdf]

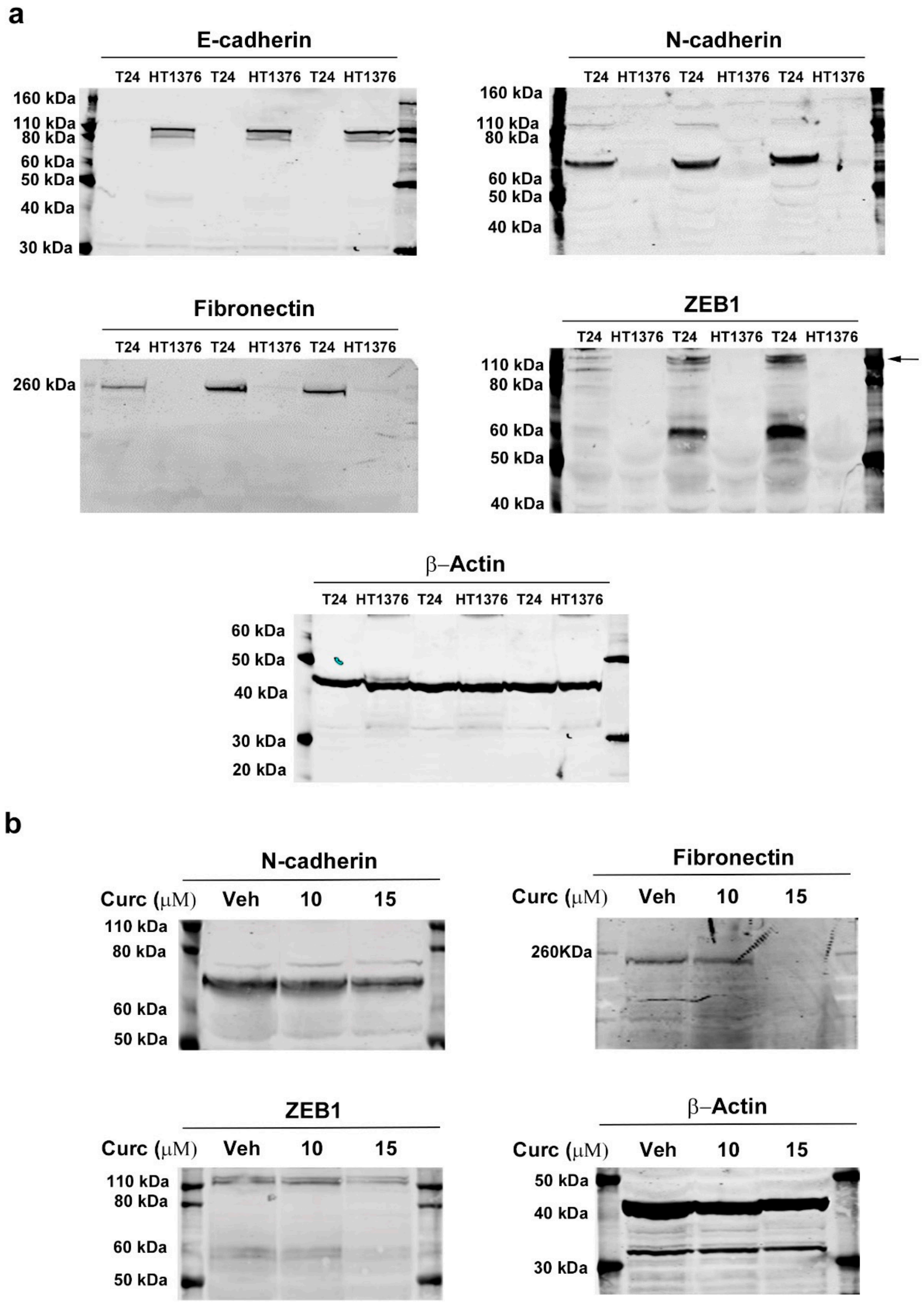

Figure S1. Non-cropped western blots from Figure 4 **(a)** and Figure 5 **(b).**

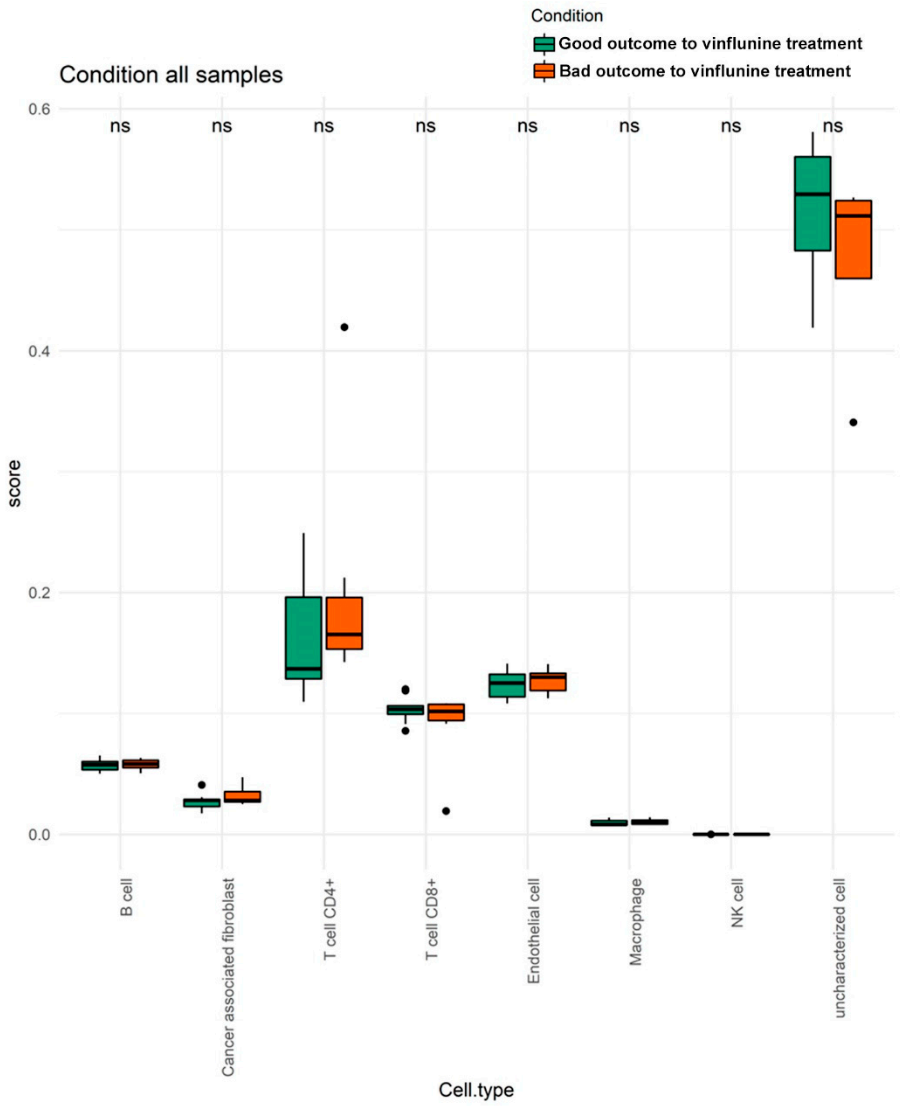

Figure S2. **Box plot depicting the distribution of different cell types scores.** For each cell type obtained and each studied condition, a box plot depicting the distribution of the scores was plotted. Statistical differences between patients with good and poor outcomes were assessed with the Student's t-test (Wilcoxon rank sum test). ns= not significant.

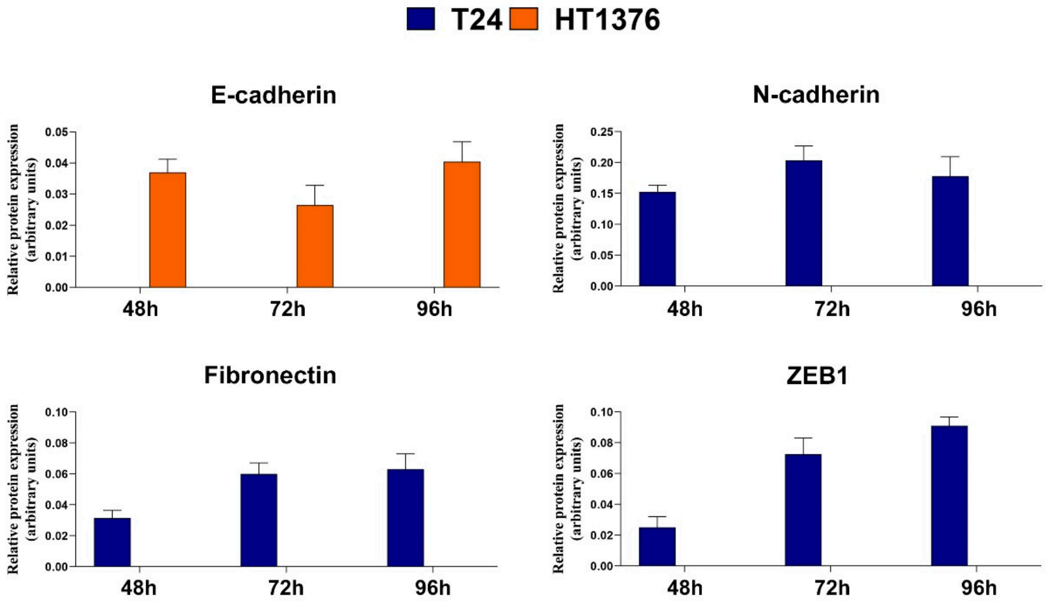

Figure S3. Semi-quantitative analysis for the Western Blot from Figure 4a.
